# Rheumatic Heart Disease in East Africa: A Systematic Review and Meta-Analysis

**DOI:** 10.1155/2023/8834443

**Published:** 2023-09-19

**Authors:** Guesh Mebrahtom, Abrha Hailay, Woldu Aberhe, Kidane Zereabruk, Teklehaimanot Haile

**Affiliations:** ^1^Department of Adult Health Nursing, School of Nursing, Aksum University, Aksum, Ethiopia; ^2^Department of Maternity and Neonatal Nursing, School of Nursing, Aksum University, Aksum, Ethiopia

## Abstract

**Background:**

Despite being a grave problem, there is little information on rheumatic heart disease's prevalence in East Africa. Therefore, the purpose of this systematic review and meta-analysis was to estimate the pooled prevalence of rheumatic heart disease in East Africa.

**Materials and Methods:**

A computerized systematic search of using multiple database searching engines was performed in search of relevant English articles from the inception of the databases to December 2019. It was done in accordance with the preferred reporting items for systematic review and meta-analysis (PRISMA) standard. The funnel plot was used to assess publication bias. R and RStudio for Windows were used for all statistical analysis. The random-effect model was used for calculating the pooled estimate of the prevalence of rheumatic heart disease.

**Results:**

The database search retrieved 1073 papers, and 80 articles (78 cross-sectional and two cohort study designs) with a total of 184575 individuals were found to be appropriate for the review. In East Africa, the overall prevalence of rheumatic heart disease was 14.67% (95% CI: 13.99% to 15.35%). In Ethiopia, Uganda, Tanzania, and Sudan, respectively, the subgroup analysis of rheumatic heart disease pooled prevalence was 22% (95% CI: 13% to 36%), 11% (95%t CI: 5% to 20%), 9% (95%t CI: 5% to 16%), and 3% (95%t CI: 1% to 10%), while the pooled prevalence of rheumatic heart disease in adults was 20% (95% CI: 12% to 30%), and in children, it was 4% (95% CI: 2% to 8%).

**Conclusions:**

From this report, the prevalence of rheumatic heart disease in East Africa is very high, affecting about one in seven people. Therefore, future strategies should emphasize preventive measures at appropriate times to minimize the burden of this type of preventable heart disease.

## 1. Introduction

Rheumatic heart disease (RHD) is the long-term consequence of acute rheumatic fever (ARF) and the autoimmune response to group A streptococcal pharyngitis, which is the leading cause of morbidity and mortality among children and young adults in developing and low-income countries [1, 2]. The 2004 World Health Organization (WHO) report and 2005 publication on the global burden of group A streptococcal disease estimated the global prevalence of RHD to be 15.6 million, which included approximately 1.01 million children in sub-Saharan Africa [3]. This estimate of the global RHD population has been doubled to 32.9 million according to the 2013 Global Burden of Disease (GBD) study [4], which accounts for at least 345,000 annual deaths [1].

Group A beta-hemolytic streptococcal infection of the upper respiratory tract, particularly pharyngitis, is the most significant avoidable risk factor for rheumatic heart disease [5]. Socioeconomic and environmental factors such as poor housing, undernutrition, overcrowding, and poverty are well-known contributors to the incidence, magnitude, and severity of rheumatic fever and rheumatic heart disease [6]. Rheumatic heart disease affects the physical, financial, and psychological well-being of individuals [6, 7]. Rheumatic heart disease continues to be a significant, preventable cause of cardiovascular death and disability in the developing countries [8].

In Africa, including East Africa, where rheumatic heart disease is an endemic leading cause of cardiovascular morbidity and mortality, at least 1.11 million children are currently estimated to have latent or confirmed rheumatic heart disease [9]. Rheumatic heart disease (RHD) is still a major public health issue, and it persists throughout Africa largely due to poverty and a weak primary health care system [7]. With little data on the prevalence of such a disabling, avoidable cardiac disorder, which additionally causes an enormous external load on health care expenses, rheumatic heart disease has continued to be the primary health concern in Africa, including Eastern African nations [10]. Therefore, in East African nations, conducting research and estimating the prevalence of rheumatic heart disease are necessary for reducing the burden of this disease. Yet, many studies have been conducted to assess the magnitude of RHD among East African countries, but reports from these countries have been inconsistent, ranging from 27/10000 to 92% [11, 12]. Additionally, no previous study had estimated the overall prevalence of rheumatic heart disease among populations in East Africa. Hence, having an overall prevalence will help to overcome those discrepancies and to have a common understanding. Therefore, the purpose of this systematic review and meta-analysis was to figure out the overall prevalence of rheumatic heart disease in Eastern Africa.

## 2. Materials and Methods

### 2.1. Setting

The nations in Eastern Africa where this systematic review and meta-analysis was conducted included Djibouti, Eritrea, Ethiopia, Kenya, Madagascar, Malawi, Rwanda, Sudan, Tanzania, and Uganda [13].

### 2.2. Search Strategy and Information Sources

From inception to December 2019, articles in English were identified using a comprehensive search approach that included PubMed/MEDLINE, Embase, Google Scholar, Web of Science, Cochrane Library, Africa Wide Information, Africa Index Medicus, Scopus, Africa journal online, and the World Health Organization (WHO) Afro Library. The literature search approach was employed using the headings of the medical subject headings (MeSH), and the MEDLINE/PubMed (AND/OR) database was used. A combination of key terms, including “rheumatic heart disease,” “acute rheumatic fever,” “valvular heart diseases,” “nation's name,” “systematic review,” and/or protocols had been used. The presence of precursor systematic reviews and/or protocols on the topic of interest was checked by searching different databases for systematic reviews. The databases searched include the international database of prospectively registered systematic reviews (PROSPERO) in the National Institute for Health and Care Research (NIHR), the Cochrane database of systematic reviews, the Joanna Briggs Institute database of systematic reviews and implementation reports (JBI-DSRIR), health technology assessment (HTA), the Campbell collaboration library, and evidence for policy and practice information (EPPI-center). There were no systematic reviews or protocols on the subject of interest, according to the search of the aforementioned databases. The reference lists of all identified articles were further searched for further articles.

### 2.3. Data Extraction, Selection, and Process

Prior to the screening and selection process, reviewers prepared a tool according to the inclusion and exclusion criteria. Before data extraction started, the tool had been piloted and revised. The search results were first uploaded to EndNote software in order to remove duplicates. Two independent reviewers extracted the data using an established and standardized collection form. For the purpose of identifying possibly eligible articles, the two reviewers (GM and KZ) independently reviewed the titles, abstracts of all retrieved citations, and the full-text search results.

Reviewers have been notified if more data is needed to verify eligibility studies. Disagreements between the reviewers were resolved by discussion with the help of a third independent reviewer (HA). When there was missing information, the reviewers got in touch with the corresponding author to ask for data. Before dropping an article, a maximum of three emails were sent to the corresponding author of the retrieved studies to request more information. For studies that occur in multiple publications, we took into account the most recent, comprehensive, and with the greatest sample size. For surveys that appear in a single article with several surveys done at various times, we treated each survey as a separate study. Information about the publication's year, country, authors, cases, total/sample size, study setting/area, study design, RHD diagnosis method, population, response rate, and prevalence or incidence of RHD were all included in the data extraction format.

### 2.4. Criteria for considering Studies for the Review

#### 2.4.1. Inclusion Criteria

*Design:* all observational studies.

*Population:* any age group in the population participated in the study.

*Publication status:* published and unpublished.

*Settings:* both community and health institution-based studies conducted in Eastern African nations.

*Language:* the only articles considered in this review were those written in English. This is due to the ease with which one is able to read and understand other languages.

*Publication or report year:* it is preferred that this study will include the past 5 to 20 years for systematic review and meta-analysis. However, due to the limitation of literatures and to look at the trend of pooled prevalence of RHD, we reviewed all publications until December 2019.

*Method of diagnosis:* all studies used different types of diagnostic criteria, like the World Health Organization (WHO) or World Heart Federation (WHF), echocardiography, the American Heart Association, clinical auscultation and/or electrocardiogram (ECG), and/or radiography, to diagnose rheumatic heart disease.

*Outcome:* prevalence of rheumatic heart disease.

#### 2.4.2. Exclusion Criteria

The following research are excluded from consideration: case report and case series studies, as well as any other studies that lacked pertinent information required to estimate the prevalence of rheumatic heart disease.

### 2.5. Risks of Bias and Quality Assessment of the Included Studies

Two reviewers (GM and WA), working independently and blindly, assessed the quality of the methodology and risk of bias. The reviewers maintained the blinding reviewing method using the Covidence software, which permits or requires each reviewer to operate without being aware of the other reviewer's option. This helps reduce errors and the risk of bias during the study selection process. Discrepancies between the reviewers were settled through dialogue and, where necessary, involving a third reviewer.

We estimated the precision (C) or margin of error for each included study, taking into account the sample size (SS) and the prevalence (*p*) of RHD observed from the following formula:

(1) $$\begin{array}{c} \text{SS}=\frac{z^{2}\times p\times \left(1-p\right)}{d^{2}}, \end{array}$$

where *Z* was the *z* value fixed at 1.96 across studies (corresponding to 95% confidence interval). The desirable margin of error is 5% (0.05) or lower.

Methodological quality was measured using the Newcastle-Ottawa scale for the included studies. The Newcastle-Ottawa scale was designed to assess the quality of meta-analyses of nonrandomized studies. This scale is primarily formulated by a star allocation system, which assigns a maximum of 10 stars for the risk of bias in three areas: a selection of study groups (4 or 5 stars), comparability of groups (2 stars), and determination of the outcome of interest or exposure (3 stars). No validation study provides a cut-off score for grading low-quality studies; a priori, we have arbitrarily established that 0–3, 4–6, and 7–10 stars would be considered at high, moderate, and low bias risk, respectively [14].

### 2.6. Data Analysis and Presentation of Results

This review has been registered in the international prospective register of reviews (PROSPERO) with the registration number (CRD42020184085), and the preferred reporting items for systematic reviews and meta-analysis (PRISMA) guidelines have been used to report the result (file 1) [15]. R 3.5.3 and RStudio 1.2.5003 were both used as data analysis software. Forest plots were drawn in order to calculate the pooled prevalence of rheumatic heart disease (RHD) and the degree of statistical heterogeneity between studies. Statistical heterogeneity was assessed using the standard chi-squared test (Cochrane's *Q* test) and quantified by calculating the *I*^2^ statistic (with values of 25%, 50%, and 75% being representative of low, medium, and high heterogeneity, respectively) [16]. There was clinical heterogeneity between the included studies. Consequently, we used a random-effect meta-analysis to estimate the overall pooled prevalence of rheumatic heart disease in Eastern Africa. The 95% confidence interval (CI) was used to explain the results, and funnel plot and Egger's regression test methods were used to assess possible publication bias. A *p* value of 0.05 indicated the presence of significant publication bias [17].

## 3. Results

### 3.1. Screening Flow

**Figure 1:**
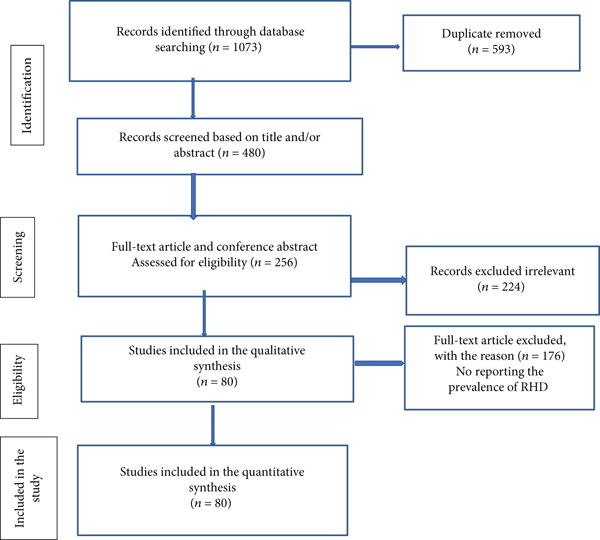
Flow chart diagram showing the selection of articles for systemic review and meta-analysis of rheumatic heart disease in East Africa, 2020.

A schematic representation of the process that was used to identify and select the studies that were included is illustrated in Figure 1. The included databases and number of included studies thereof were PubMed (464), Google Scholar (193), Cochrane Library (39), Africa Wide Information (23), the World Health Organization (WHO) Afro Library (19), African Journal Online (212), Web of Science (43), Scopus (65), and African Index Medicus (15). Based on the predefined criteria and quality assessment, 593 duplicates were identified and removed. Subsequently, we screened 480 titles and abstracts and excluded 224 irrelevant papers. Then, of the 256 articles and conference abstracts assessed for eligibility criteria, 176 did not report a prevalence of RHD. In the end, this systematic review and meta-analysis included 80 full-text articles and 184575 participants of all ages. The detailed steps of the screening process are shown below in a PRISMA flow chart of the study selection.

### 3.2. Study Characteristics

The meta-analysis included a total of 78 cross-sectional and two cohort studies. More than one-third (33, 41.25%) of the studies were conducted in Ethiopia, followed by (12, 15%) in Sudan and (12, 15%) in Uganda, five (6.25%) from Kenya, four from Rwanda (5%), four (5%) from Tanzania, three (3.75%) from Madagascar, three (3.75%) from Malawi, two (2.5%) from Djibouti, and two (2.5%) from Eritrea. The quality of each primary study assessed using the Newcastle-Ottawa scale shows no significant risk. Thus, there was a low chance of bias in any of the included studies that were taken for consideration in this systematic review and meta-analysis (Table 1).

### 3.3. The Pooled Prevalence of Rheumatic Heart Disease and Its Trends in East Africa

The rheumatic heart disease pooled prevalence in East Africa was 14.67% (95% CI: 13.99% to 15.35%), and the inverse variance (*I*^2^) was 100%, indicating heterogeneity in the reported prevalence of RHD among the included studies (appendix 2). This heterogeneity could be a result of differences in the diagnostic or screening criteria for RHD used by the included studies. A leave-one-out sensitivity analysis was carried out to see whether the findings of a single study had a significant impact on the pooled prevalence of RHD in East Africa. However, all the results of this sensitivity analysis were within the 95% CI limits of the pooled prevalence (13.99 to 15.35%), indicating that no significant study may have had an impact on the observed pooled prevalence of RHD (appendix 3).

**Figure 2:**
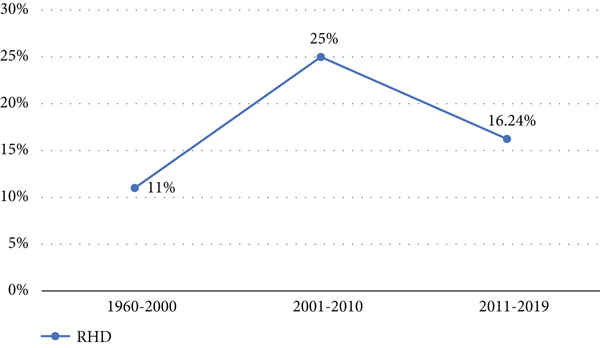
Trend of rheumatic heart disease pooled prevalence in East Africa.

**Figure 3:**
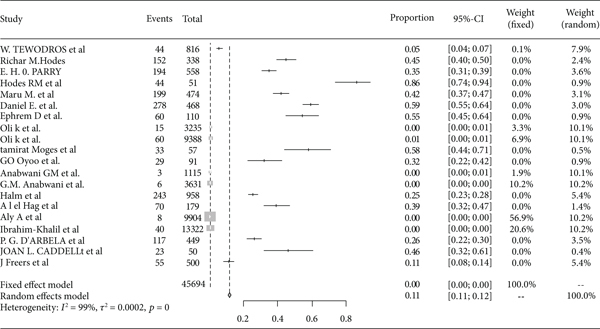
Forest plot of rheumatic heart disease prevalence in East Africa from 1960 to 2000.

**Figure 4:**
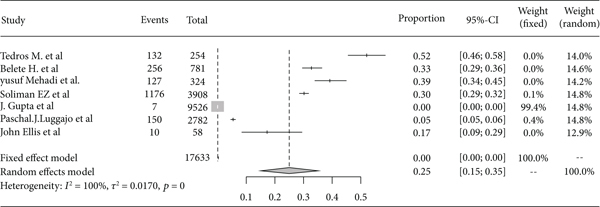
Forest plot of the prevalence of rheumatic heart disease in East Africa from 2001 to 2010.

The pooled prevalence trends of rheumatic heart disease were decreasing over the last nine years (2011–2019) (Figure 2). The pooled prevalence of rheumatic heart disease in East Africa from 1960 to 2000 was 11% (95% CI: 11% to 12%) (Figure 3), and from 2001 to 2020 it was 25% (95% CI: 15% to 35%) (Figure 4).

**Figure 5:**
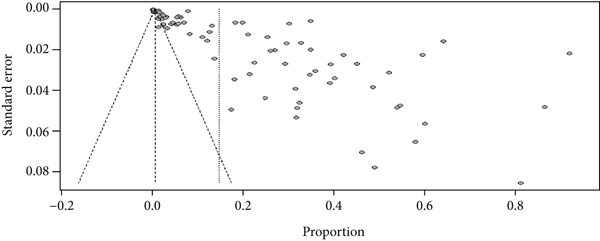
Funnel plot indicating the presence of publication bias.

Because the funnel plot showed an asymmetrical distribution (Figure 5), we conducted Egger's regression test, which was found to be significant with a *p* value of 0.00, which indicates evidence of publication bias in the included studies. Hence, there are unpublished articles that could modify the pooled prevalence of rheumatic heart disease.

### 3.4. Subgroup Analysis of Rheumatic Heart Disease Prevalence by Study Countries in East Africa

**Figure 6:**
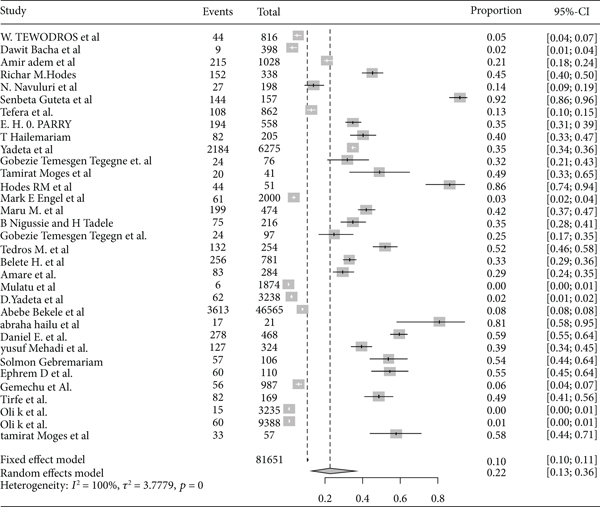
Forest plot of the pooled prevalence of rheumatic heart disease in Ethiopia.

**Figure 7:**
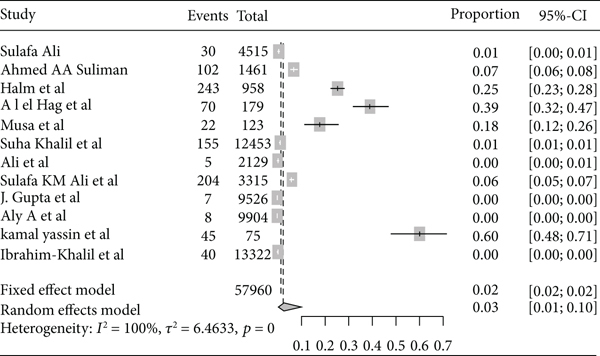
Forest plot of the pooled prevalence of rheumatic heart disease in Sudan.

**Figure 8:**
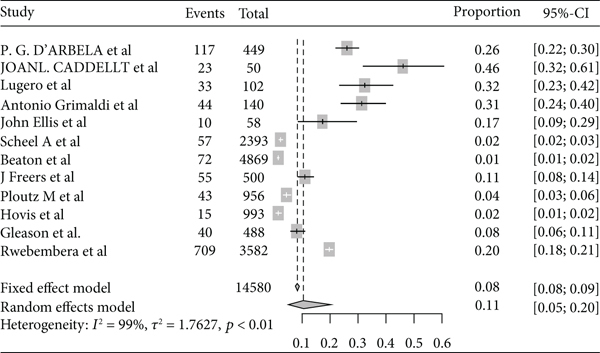
Forest plot of the pooled prevalence of rheumatic heart disease in Uganda.

**Figure 9:**
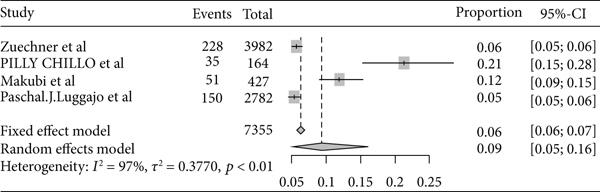
Forest plot of the pooled prevalence of rheumatic heart disease in Tanzania.

Based on the subgroup analysis of rheumatic heart disease by the study country, the pooled point estimate prevalence of rheumatic heart disease in Ethiopia was 22% (95% CI: 13% to 36%) (Figure 6); in Sudan, it was 3% (95% CI: 1% to 10%) (Figure 7); in Uganda, it was 11% (95% CI: 5% to 20%) (Figure 8); and in Tanzania, it was 9% (95% CI: 5% to 16%) (Figure 9).

### 3.5. Subgroup Analysis of the Prevalence of Rheumatic Heart Disease in East Africa among Adults and Children

**Figure 10:**
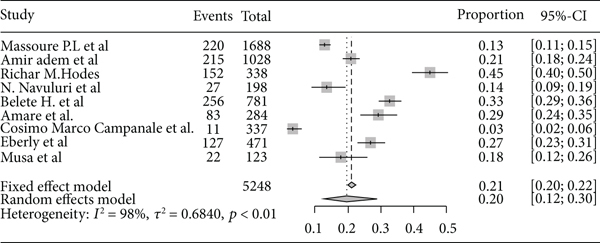
Forest plot of the pooled prevalence of rheumatic heart disease among adults in East Africa.

**Figure 11:**
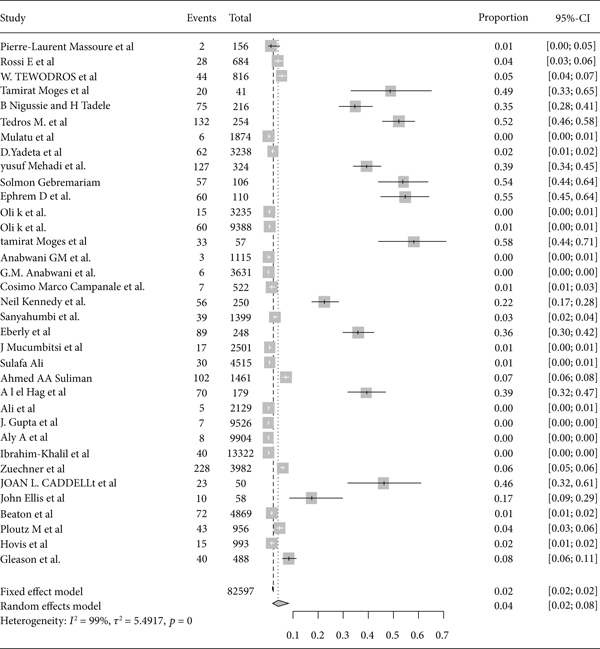
Forest plot of the pooled prevalence of rheumatic heart disease among children in East Africa.

Based on the subgroup analysis of rheumatic heart disease among adults and children in East Africa, the pooled point estimate prevalence of rheumatic heart disease among the adult population was 20% (95% CI: 12% to 30%) (Figure 10), and among children, it was 4% (95% CI: 2% to 8%) (Figure 11).

## 4. Discussion

Rheumatic heart disease is a preventable yet serious public health problem in low- and middle-income countries and in marginalized communities in high-income countries, including indigenous populations, where poverty is prevalent, people are living in overcrowded conditions, poor housing, and undernutrition, and access to health services is limited [6, 92]. Furthermore, studies addressing the prevalence of rheumatic heart disease among populations in East Africa are limited. Thus, the purpose of this systematic review and meta-analysis was to estimate the pooled prevalence of rheumatic heart disease in East Africa. It focuses on the epidemiology of rheumatic heart disease in East African nations to acquire a better understanding of the medical condition and mitigate the burden of rheumatic heart disease.

A total of eighty studies were included in the final analysis, providing an overall pooled prevalence of rheumatic heart disease in East Africa of 14.67% (95% CI: 13.99% to 15.35%). This is lower than a global systematic review and meta-analysis of population-based echocardiographic study, which reported 26.1% [93], and this discrepancy could be explained by a difference in definition for case detection and diagnostic criteria for rheumatic heart disease [92, 94].

However, this research result is higher than a systematic review and meta-analysis of prevalence among children and adolescents which reported that the pooled prevalence of rheumatic heart disease detected by cardiac auscultation was 2·9 per 1000 people, and by echocardiography, it was 12·9 per 1000 people [95]. This discrepancy could be explained by differences in definition for case detection and diagnostic criteria for rheumatic heart disease, in the living environment, in easily accessible health care, in early treatment and vaccination for group A streptococcal throat infection prevention and awareness, in the socioeconomic status of the populations, in the duration of studies, and in immune and genetic factors [92, 94, 96–99].

The prevalence of rheumatic heart disease in the studies included in this systematic review and meta-analysis varied, ranging from 27/10000 by Anabwani and Bonhoeffer [11] in Kenya to 92% by Guteta et al. [12] in Ethiopia, respectively. This discrepancy could be due to differences in the objectives of these studies, a variation in the diagnostic assessment, or a variation in the duration of the studies. Additionally, to look at the pattern of rheumatic heart disease prevalence in East Africa, we analyzed RHD pooled prevalence in year intervals and found that rheumatic heart disease prevalence has declined in the last years (2011–2019). This might be due to increased health care services and the health-seeking behavior of the communities. Another possible explanation might be the increased interventions carried out to reduce the prevalence of rheumatic heart disease in Africa, including East Africa. In 2015, the social cluster of the Africa Union Commission hosted a consultation with rheumatic heart disease experts in Addis Ababa, Ethiopia, to develop a roadmap for eliminating rheumatic heart disease in Africa [100]. Hence, this might increase awareness of primary and secondary prevention and early detection of rheumatic heart disease.

To look at the heterogeneity of the studies included in this meta-analysis, we considered subgroup analysis of the prevalence of rheumatic heart disease in East Africa among adult and children's populations. Hence, the subgroup analysis of rheumatic heart disease pooled prevalence among the adult population in East Africa was 20% (95% CI: 12% to 30%), which is higher than the prevalence of chronic rheumatic heart disease among Chinese adults, which was reported at 186/100,000, or 2 out of 1000 adults [101], and the overall pooled prevalence of rheumatic heart disease in East Africa. This could be explained by the fact that more than half of those studies among adults were conducted in Ethiopia, which is the country with the highest pooled prevalence of rheumatic heart disease in our study. Also, most of those studies were conducted in hospital settings, which might have better diagnostic tools and services than communities.

While the subgroup analysis of the prevalence of rheumatic heart disease among East African children was 4% (95% CI: 2% to 8%), which is lower than the echocardiographic prevalence of rheumatic heart disease among Brazilian schoolchildren (42/1000) [102] and the overall pooled prevalence of rheumatic heart disease in East Africa but higher than the study conducted in Lagos, which reported 1.1 per 10,000 children [103], this variation could be explained by the higher prevalence of rheumatic heart disease found among adults who might visit health care after developing complications due to lower awareness of prevention and early health care-seeking behavior in low socioeconomic status populations like Ethiopians. Also, most of the studies among children were conducted in a school community where the symptomatic and sick children might not come to school due to differences in definition for case detection and diagnostic criteria for rheumatic heart disease, in the living environment, in the duration of studies, as well as in immune and genetic factors [92, 98, 99, 104].

Also, we considered country-wise subgroup analyses of rheumatic heart disease among four East African countries and found that 22% (95% CI: 13% to 36%), 11% (95% CI: 5% to 20%%), 9% (95% CI: 5% to 16%), and 3% (95% CI: 1% to 10%%) of the pooled prevalence of rheumatic heart disease were in Ethiopia, Uganda, Tanzania, and Sudan, respectively. This discrepancy might be explained by differences in the definition of case detection and diagnostic criteria in study settings, as most of the studies in Ethiopia were conducted in hospital settings where the number of cases detected might be higher compared to school as well as community settings; a difference in the number of studies since more than one-third of the studies were from Ethiopia; a variation in the duration of studies; a difference in living environments; and immune and genetic factors. Also, low socioeconomic status, low access to health care, and low awareness of the prevention of rheumatic heart disease are common among Ethiopians compared to Sundaneses, Ugandans, and Tanzanians [52, 100].

Generally, there is no use of standardized assessment requirements or diagnostic procedures in all health care settings of clinical practice, or specific clinicians are using their particular clinical expertise in the diagnosis and examination of patients for rheumatic heart disease. Our meta-analysis findings have implications in clinical practice that help pay attention to the prevention and care of rheumatic heart disease patients. This pooled estimate point for rheumatic heart disease provides updated evidence to advance the prevention strategy and serves as a key health and safety indicator. Finding the prevalence of rheumatic heart disease may provide guidance on preventions, such as improving living conditions, reducing overcrowding, increasing awareness on the prevention of rheumatic heart disease, vaccination, and early detection and treatment of group A streptococcal throat infections. The significance of this study, especially the profound difference between countries and studies, reflects screening and evaluation of rheumatic heart disease, which require the establishment of standard evaluation tools and diagnostic criteria in both community and hospital settings.

## 5. Conclusions

According to our study results, one in seven people in East Africa suffers from rheumatic heart disease. This finding implies that rheumatic heart disease is considerably more common in East Africa. The country's Ministry of Health, health policymakers, WHO, clinicians, and other health care providers should pay attention to strengthening rheumatic heart disease preventive measures. In Eastern African countries, we suggest that more attention be given to addressing the profound consequences of rheumatic heart disease. We recommended strengthening preventive measures, such as enhancing living conditions, reducing overcrowding, raising awareness of the prevention of rheumatic heart disease, nutrition, vaccination, early detection and treatment of group A streptococcal throat infections, and establishing country-based interventions, because rheumatic heart disease is preventable.

## 6. Limitations of This Study

This systematic review and meta-analysis draw up an overview of the pooled prevalence of rheumatic heart disease in East African countries. However, the findings of this study could have certain limitations. Among the limitations, subgroup analysis for studies between all countries was difficult to carry out due to statistical constraints and a limited number of studies, which is why we only conducted subgroup analysis for Ethiopia, Uganda, Tanzania, and Sudan. This makes our review subject to a high degree of heterogeneity between studies, which could affect the meta-analysis results as we do not report from all countries of the study. But the model of random effects was used to achieve the pooled results that minimize this heterogeneity among studies. In addition, methodological variations in assessments and diagnosis of RHD among studies included in this work could also affect meta-analysis results with extensive clinical heterogeneity across studies. Publication bias was also another limitation of our review.

## Figures and Tables

**Table 1:** Characteristics of studies included in the systematic review and meta-analysis of the prevalence of RHD in East Africa, 2020.

Author	Country	Diagnostic method	Year	SD	Cases	Total	PR	RR	Area/setting	Population	NOS
Massoure et al. [18]	Djibouti	Echocardiography	2013	cs	220	1688	13%	100%	Hospital	Adult population	7
Massoure et al. [19]	Djibouti	Echocardiography	2013	cs	2	156	1.28%	100%	Hospital	Children	7
Rossi et al. [20]	Eritrea	Echocardiography	2014	cs	28	648	4%	100%	School	Children	9
Otto et al. [21]	Eritrea	Echocardiography	2011	cs	8	348	2.3%	100%	Hospital	Pregnant	9
Tewodros et al. [22]	Ethiopia	Echocardiography	1992	cs	44	816	5.4%	100%	Hospital	Children	9
Bacha et al. [23]	Ethiopia	Echocardiography	2019	cs	9	398	2.3%	100%	Hospital	Pregnant	9
Adem et al. [24]	Ethiopia	Echocardiography	2014	cs	215	1028	21%	100%	Hospital	Adult population	9
Hodes [25]	Ethiopia	Echocardiography	1988	cs	152	338	45%	100%	Hospital	Adult population	8
Navuluri et al. [26]	Ethiopia	Echocardiography	2016	cs	27	198	13.6%	100%	Hospital	Adult population	8
Guteta et al. [12]	Ethiopia	Echocardiography	2016	cs	144	157	92%	100%	Hospital	General population	7
Tefera et al. [27]	Ethiopia	Echocardiography	2017	cs	108	862	11.80%	84%	Hospital	General population	9
Parry and Gordon [28]	Ethiopia	Echocardiography	1968	cs	194	558	34.8%	100.00%	Hospital	General population	8
Hailemariam [29]	Ethiopia	Echocardiography	2014	cs	82	205	40%	100%	Hospital	General population	8
Yadeta et al. [30]	Ethiopia	Echocardiography	2017	cs	2184	6275	34.6%	88%	Hospital	General population	8
Tegegne et al. [31]	Ethiopia	Echocardiography	2014	cs	24	76	31.6%	100%	Hospital	General population	8
Moges et al. [32]	Ethiopia	Echocardiography	2015	cs	20	41	49%	100%	Hospital	Children	9
Hodes [33]	Ethiopia	Clinical auscultation and radiography	1993	cs	44	51	86.2%	100%	Hospital	General population	7
Engel [34]	Ethiopia	Echocardiography	2015	cs	61	2000	3.05%	100%	Hospital	General population	7
Maru [35]	Ethiopia	Echocardiography	1993	cs	199	474	42%	100%	Hospital	General population	8
Nigussie and Tadele [36]	Ethiopia	Echocardiography	2019	cs	75	216	34.72%	100%	Hospital	Children	8
Tegegne et al. [37]	Ethiopia	Echocardiography	2015	co	24	97	24.74%	100%	Hospital	General population	8
Malede and Haileamlak [38]	Ethiopia	Echocardiography	2006	cs	132	254	52%	100%	Hospital	Children	9
Habte et al. [39]	Ethiopia	Echocardiography	2010	cs	256	781	32.80%	93.30%	Hospital	Adult population	9
Amare et al. [40]	Ethiopia	Echocardiography	2015	cs	83	284	29.20%	100%	Hospital	Adult population	9
Mulatu et al. [41]	Ethiopia	Echocardiography	2015	cs	6	1874	0.32%	100%	Hospital	Children	9
Yadeta et al. [42]	Ethiopia	Echocardiography	2016	cs	62	3238	1.90%	100%	School	Children	8
Bekelea et al. [43]	Ethiopia	Echocardiography	2015	cs	3613	46565	7.76%	100%	Hospital	General population	9
Hailu et al. [44]	Ethiopia	Echocardiography	2019	cs	17	21	81%	100%	Hospital	Pregnancy	9
Daniel and Abegaz[45]	Ethiopia	Echocardiography	1992	cs	278	468	59.40%	100%	Hospital	Children	7
Mehadi et al. [46]	Ethiopia	Echocardiography	2004	cs	127	324	39.20%	100%	Hospital	Children	7
Gebremariam and Moges [47]	Ethiopia	Echocardiography	2016	cs	57	106	53.70%	100%	Hospital	Children	9
Ephrem et al. [48]	Ethiopia	Echocardiography	1990	cs	60	110	54.50%	100%	Hospital	Children	7
Gemechu et al. [49]	Ethiopia	Echocardiography	2016	cs	56	987	5.67%	100%	Rural community	General population	7
Tirfe et al. [50]	Ethiopia	Echocardiography	2020	co	82	169	48.5%	100%	Hospital	General population	9
Oli et al. [51]	Ethiopia	Clinical auscultation and radiography	1992	cs	15	3235	0.46%	100%	Rural community	Children	8
Oli and Porteous [52]	Ethiopia	Echocardiography	1999	cs	60	9388	0.64%	93%	Community	Children	9
Moges [53]	Ethiopia	Echocardiography	1999	cs	33	57	56.10%	100%	Hospital	Children	9
Rebecca [54]	Kenya	Echocardiography	2016	cs	580	906	64%	86.30%	Hospital	General population	8
Oyoo and Ogola [55]	Kenya	Echocardiography	1999	cs	29	91	32%	100%	Hospital	General population	8
Koech et al. [56]	Kenya	American heart association guidelines	2012	cs	582	3196	18%	100%	Hospital	General population	9
Anabwani and Bonhoeffer [11]	Kenya	Echocardiography	1996	cs	3	1115	0.0027%	100%	Hospital	Children	9
Anabwani et al. [57]	Kenya	Clinical auscultation	1989	cs	6	3631	0.17%	97%	School	Children	9
Campanale et al. [58]	Madagascar	Echocardiography	2017	cs	18	859	2.10%	100%	Hospital and school	General population	9
Campanale et al. [58]	Madagascar	Echocardiography	2017	cs	7	522	1.4%	100%	Hospital and school	Children	9
Campanale et al. [58]	Madagascar	Echocardiography	2017	cs	11	337	3.3%	100%	Hospital and school	Adult population	9
Kennedy and Miller [59]	Malawi	Echocardiography	2013	cs	56	250	22.4%	100%	Hospital	Children	9
Soliman and Juma [60]	Malawi	Echocardiography	2008	cs	1176	3908	30.10%	100%	Hospital	General population	8
Sanyahumbi et al. [61]	Malawi	Echocardiography	2016	cs	39	1399	3.40%	96.50%	School	Children	8
Eberly et al. [62]	Rwanda	Echocardiography	2018	cs	212	719	29.50%	100%	Hospital	General population	8
Eberly et al. [62]	Rwanda	Echocardiography	2018	cs	89	248	36%	100%	Hospital	Children	8
Eberly et al. [62]	Rwanda	Echocardiography	2018	cs	127	471	27%	100%	Hospital	Adult population	8
Mucumbitsi et al. [63]	Rwanda	Echocardiography	2017	cs	17	2501	0.68%	93%	Hospital	Children	8
Ali et al. [64]	Sudan	Echocardiography	2018	cs	30	4515	1%	100%	School	Children	9
Kafle and Alurkar[65]	Sudan	Echocardiography	2011	cs	102	1461	7%	100%	Hospital	Children	9
Halim and Jacques [66]	Sudan	Clinical auscultation radiologic, and ECG	1960	cs	243	958	25%	100%	Hospital	General population	9
El Hag [67]	Sudan	Echocardiography	1994	Cs	70	179	39%	100%	Hospital	Children	9
Musa et al. [68]	Sudan	Echocardiography	2018	cs	22	123	17.89%	100%	Hospital	Adult population	8
Khalil et al. [69]	Sudan	Echocardiography	2015	cs	155	12453	1.24%	98.6%	Hospital	General population	7
Ali et al. [70]	Sudan	Echocardiography	2018	cs	5	2129	0.23%	100%	School	Children	9
Yadav et al. [71]	Sudan	Echocardiography	2010	cs	7	9526	0.07%	96.40%	School	Children	9
Ali et al. [72]	Sudan	Echocardiography	2017	cs	204	3315	6.20%	100%	Community	General population	9
Hasab et al. [73]	Sudan	Echocardiography	1997	cs	8	9904	0.08%	100%	School	Children	9
Yassin et al. [74]	Sudan	Echocardiography	2015	cs	45	75	60%	100%	Hospital	Pregnancy	9
Ibrahim-Khalil et al. [75]	Sudan	Clinical auscultation and radiographic X-ray	1992	cs	40	13322	0.30%	100%	School	Children	9
Zuechner et al. [76]	Tanzania	Echocardiography	2019	cs	228	3982	6%	100%	Hospital	Children	9
Mmbali and Chillo [77]	Tanzania	Echocardiography	2017	cs	35	164	21.40%	100%	Hospital	General population	9
Makubi et al. [78]	Tanzania	Echocardiography	2014	cs	51	427	12%	82%	Hospital	General population	7
Luggajo [79]	Tanzania	Echocardiography	2009	cs	150	2782	5.40%	100%	Hospital	General population	8
d'Arbela et al. [80]	Uganda	Clinical auscultation and radiography	1966	cs	117	449	26%	100%	Hospital	General population	8
Caddell et al. [81]	Uganda	Clinical auscultation and radiography	1966	cs	23	50	46%	100%	Hospital	Children	8
Lugero et al. [82]	Uganda	Echocardiography	2016	cs	33	102	32%	100%	Hospital	General population	7
Grimaldi et al. [83]	Uganda	Echocardiography	2014	Cs	44	140	31%	100%	Hospital	General population	7
Ellis et al. [84]	Uganda	Echocardiography	2007	cs	10	58	17%	100%	Hospital	Children	7
Scheel et al. [85]	Uganda	Echocardiography	2018	cs	57	2393	2.45%	98%	Community	General population	9
Beaton et al. [86]	Uganda	Echocardiography	2013	cs	72	4869	1.50%	97%	School	Children	9
Freers et al. [87]	Uganda	Echocardiography	1993	cs	55	500	11%	100%	Hospital	General population	9
Ploutz et al. [88]	Uganda	Echocardiography	2015	cs	43	956	4.50%	95.40%	School	Children	9
Hovis et al. [89]	Uganda	Echocardiography	2019	cs	15	993	1.50%	100%	Hospital	Children	9
Gleason et al. [90]	Uganda	Echocardiography	2016	cs	40	488	0.82%	99.60%	Hospital	Children	8
Rwebembera et al. [91]	Uganda	Echocardiography	2018	cs	709	3582	19.80%	100%	Hospital	General population	9

ECG: electrocardiogram; CS: cross-sectional; CO: cohort; SD: study design; PR: prevalence rate; RR: response rate; RHD: rheumatic heart disease; NOS: Newcastle-Ottawa scale.

## Data Availability

The data analyzed during the current meta-analysis is available from the corresponding author on a reasonable request.

## Abbreviations

CI: Confidence interval
RHD: Rheumatic heart disease
PRISMA-P: Preferred reporting items for systematic reviews and meta-analysis protocol
WHO: World Health Organization.

## Supplementary Materials

Supplementary Materials The supplementary materials for the systematic review and meta-analysis of this review include Appendix 1 for the search strategy and information sources, Appendix 2 for the forest plot of the overall pooled prevalence of RHD in East Africa, Appendix 3 for the sensitivity analysis of the included studies, File 1 for the PRISMA-P of the preferred reporting items for systematic reviews and meta-analysis protocol, and File 2 for the Newcastle-Ottawa scale for the methodological quality assessment of the included studies.
