# Supplementary material for: Rheumatic Heart Disease in East Africa: A Systematic Review and Meta-Analysis
Source: Int J Rheumatol. 2023 Sep 19;2023:8834443. doi: 10.1155/2023/8834443 (PMC10522432; doi:10.1155/2023/8834443)
Supplement: Supplementary Materials — The supplementary materials for the systematic review and meta-analysis of this review include Appendix 1 for the search strategy and information sources, Appendix 2 for the forest plot of the overall pooled prevalence of RHD in East Africa, Appendix 3 for the sensitivity analysis of the included studies, File 1 for the PRISMA-P of the preferred reporting items for systematic reviews and meta-analysis protocol, and File 2 for the Newcastle-Ottawa scale for the methodological quality assessment of the included studies. [file 8834443.f1.zip › Appendix 1.docx]

## **Search Strategy and Information Sources**

Articles were retrieved through comprehensive search strategy using (PubMed/MEDLINE, Embase, Google Scholar, Web of Science, Cochrane library, Africa Wide Information, and Africa Index Medicus, Scopus, Africa journal online, World Health Organization (WHO) Afro library) from inception to December 2019. The literature search technique was developed using the headings of the medical subject headings (MeSH), MEDLINE/PubMed (AND/OR) database was used. The combination of key terms including “Rheumatic Heart Disease”, “Acute Rheumatic Fever”,” Rheumatic Fever,” Valvular Heart Diseases”,” “Nation’s name”, “systematic review” and protocols were used. The presence of precursor systematic review and/or protocol on the topic of interest was checked via searching different databases for systematic review. The databases searched include Cochrane database of a systematic review, Joanna Briggs Institute database of a systematic review and implementation reports (JBI-DSRIR), health technology assessment-HTA, the Campbell collaboration library and evidence for policy and practice information (EPPI-center). The search from the above databases confirmed that there was no systematic review and /or protocol on the topic of interest in these Sub Saharan East African nations. All identified papers' reference lists were subsequently searched to find extra articles.

| Serial number | Databases | Number of articles found | Number of articles included | Number of Excluded articles | Reason for exclusion |
| --- | --- | --- | --- | --- | --- |
| 1 | PubMed | n=464 | n=28 | n=436 | Duplicates(n=324), Irrelevant and not reporting prevalence(n=112) |
| 2 | Google Scholar | n=193 | n= 19 | n=174 | Duplicates (n=134), Irrelevant and not reporting prevalence(n=40) |
| 3 | Cochran Library | n=39 | n=0 | n=39 | Duplicates(n=11), Irrelevant and not reporting prevalence (n=28) |
| 4 | Africa wide Information | n=23 | n=0 | n=23 | Duplicates(n=9) and Irrelevant and not reporting prevalence (n=14) |
| 5 | World Health Organization (WHO) afro library | n=19 | n=0 | n=19 | Irrelevant and not reporting prevalence (n=19) |
| 6 | African journal online | 212 | n=22 | n=190 | Duplicates(n=103)  Irrelevant and not reporting prevalence (n=87) |
| 7 | Web of Science | 43 | n=0 | n=43 | Duplicates (n=12)  Irrelevant and not  reporting prevalence (n=31) |
| 8 | Scopus | 65 | n=11 | n=54 | Irrelevant and not reporting prevalence (n=54) |
| 9 | African Index Medicus (AID) | 15 | n=0 | n=15 | Irrelevant and not reporting prevalence (n=15) |

The included databases and number of included studies thereof were PubMed (464), Google Scholar (193), Cochran Library (39), Africa wide information (23), the World Health Organization (WHO) afro library (19), African Journal online (212), Web of Science (43), Scopus (65), and African Index Medicus (15). Based on the pre-defined criteria and quality assessment, 593 duplicates were identified and removed. Subsequently, we screened 480 titles and abstracts and excluded 224 irrelevant papers. Then, 256 articles and conference abstracts assessed for eligibility criteria, 176 of those were not reported prevalence of RHD. Finally, 80 full-text articles with 184575 total participants were included in this systematic review and meta-analysis.
