# Supplementary material for: Rheumatic Heart Disease in East Africa: A Systematic Review and Meta-Analysis
Source: Int J Rheumatol. 2023 Sep 19;2023:8834443. doi: 10.1155/2023/8834443 (PMC10522432; doi:10.1155/2023/8834443)
Supplement: Supplementary Materials — The supplementary materials for the systematic review and meta-analysis of this review include Appendix 1 for the search strategy and information sources, Appendix 2 for the forest plot of the overall pooled prevalence of RHD in East Africa, Appendix 3 for the sensitivity analysis of the included studies, File 1 for the PRISMA-P of the preferred reporting items for systematic reviews and meta-analysis protocol, and File 2 for the Newcastle-Ottawa scale for the methodological quality assessment of the included studies. [file 8834443.f1.zip › Appendix 3.docx]

| **Study (Author) proportion 95%-CI** |
| --- |
| Omitting Massoure P.L et al 0.1466 [0.1398, 0.1534] |
| Omitting Pierre-Laurent Massoure et al 0.1489 [0.1420, 0.1557] |
| Omitting Rossi E et al 0.1484 [0.1416, 0.1553] |
| Omitting Havard Otto et al 0.1488 [0.1419, 0.1556] |
| Omitting W. TEWODROS et al 0.1482 [0.1413, 0.1550] |
| Omitting Dawit Bacha et al 0.1488 [0.1419, 0.1556] |
| Omitting Amir adem et al 0.1453 [0.1385, 0.1521] |
| Omitting Richar M. Hodes 0.1436 [0.1368, 0.1504] |
| Omitting N. Navuluri et al 0.1467 [0.1399, 0.1535] |
| Omitting Senbeta Guteta et al 0.1358 [0.1293, 0.1424] |
| Omitting Tefera et al. 0.1468 [0.1400, 0.1537] |
| Omitting E. H. 0. PARRY 0.1439 [0.1371, 0.1507] |
| Omitting T Hailemariam 0.1448 [0.1380, 0.1516] |
| Omitting Yadeta D et al 0.1374 [0.1311, 0.1438] |
| Omitting Gobezie Temesgen Tegegne et. al 0.1460 [0.1392, 0.1528] |
| Omitting Tamirat Moges et al 0.1460 [0.1392, 0.1528] |
| Omitting Hodes RM et al 0.1433 [0.1365, 0.1501] |
| Omitting Mark E Engel et al 0.1488 [0.1419, 0.1556] |
| Omitting Maru M. et al 0.1433 [0.1365, 0.1501] |
| Omitting B Nigussie and H Tadele 0.1451 [0.1383, 0.1519] |
| Omitting Gobezie Temesgen Tegegn et al. 0.1461 [0.1393, 0.1529] |
| Omitting Tedros M. et al 0.1435 [0.1367, 0.1503] |
| Omitting Belete H. et al 0.1438 [0.1370, 0.1505] |
| Omitting Amare et al. 0.1452 [0.1384, 0.1520] |
| Omitting Mulatu et al 0.1500 [0.1431, 0.1570] |
| Omitting D. Yadeta et al 0.1492 [0.1423, 0.1560] |
| Omitting Abebe Bekele et al 0.1427 [0.1363, 0.1492] |
| Omitting abraha hailu et al 0.1455 [0.1387, 0.1523] |
| Omitting Daniel E. et al. 0.1410 [0.1343, 0.1478] |
| Omitting yusuf Mehadi et al. 0.1442 [0.1374, 0.1510] |
| Omitting Solmon Gebremariam 0.1449 [0.1381, 0.1517] |
| Omitting Ephrem D et al. 0.1448 [0.1380, 0.1516] |
| Omitting Gemechu et Al. 0.1481 [0.1413, 0.1550] |
| Omitting Tirfe et al. 0.1445 [0.1377, 0.1513] |
| Omitting Oli k et al. 0.1502 [0.1432, 0.1571] |
| Omitting Oli k et al. 0.1511 [0.1441, 0.1581] |
| Omitting tamirat Moges et al 0.1455 [0.1387, 0.1523] |
| Omitting Rebecca H. Lumsden et al. 0.1376 [0.1309, 0.1442] |
| Omitting G O Oyoo et al. 0.1459 [0.1391, 0.1527] |
| Omitting MYRA M. KOECH et al. 0.1450 [0.1382, 0.1518] |
| Omitting Anabwani GM et al. 0.1498 [0.1429, 0.1567] |
| Omitting G.M. Anabwani et al. 0.1521 [0.1450, 0.1592] |
| Omitting Cosimo Marco Campanale et al. 0.1489 [0.1421, 0.1558] |
| Omitting Cosimo Marco Campanale et al. 0.1491 [0.1422, 0.1559] |
| Omitting Cosimo Marco Campanale et al. 0.1485 [0.1417, 0.1554] |
| Omitting Neil Kennedy et al. 0.1459 [0.1391, 0.1527] |
| Omitting Soliman EZ et al 0.1413 [0.1347, 0.1480] |
| Omitting Sanyahumbi et al. 0.1488 [0.1420, 0.1557] |
| Omitting Eberly et al 0.1443 [0.1375, 0.1511] |
| Omitting Eberly et al 0.1448 [0.1380, 0.1516] |
| Omitting Eberly et al 0.1450 [0.1382, 0.1518] |
| Omitting J Mucumbitsi et al 0.1497 [0.1428, 0.1566] |
| Omitting Sulafa Ali 0.1501 [0.1432, 0.1570] |
| Omitting Ahmed AA Suliman 0.1479 [0.1410, 0.1547] |
| Omitting Halm et al 0.1447 [0.1379, 0.1515] |
| Omitting A l el Hag et al 0.1450 [0.1382, 0.1518] |
| Omitting Musa et al 0.1464 [0.1396, 0.1532] |
| Omitting Suha Khalil et al 0.1503 [0.1434, 0.1573] |
| Omitting Ali et al 0.1505 [0.1435, 0.1574] |
| Omitting Sulafa KM Ali et al 0.1480 [0.1411, 0.1548] |
| Omitting J. Gupta et al 0.1623 [0.1542, 0.1704] |
| Omitting Aly, A et al 0.1620 [0.1539, 0.1701] |
| Omitting kamal yassin et al 0.1451 [0.1383, 0.1519] |
| Omitting Ibrahim-Khalil et al 0.1548 [0.1475, 0.1622] |
| Omitting Zuechner et al 0.1481 [0.1412, 0.1549] |
| Omitting PILLY CHILLO et al 0.1461 [0.1393, 0.1529] |
| Omitting Makubi et al 0.1469 [0.1401, 0.1538] |
| Omitting Paschal.J. Luggajo et al 0.1482 [0.1414, 0.1550] |
| Omitting P. G. D'ARBELA et al 0.1452 [0.1384, 0.1520] |
| Omitting JOAN L. CADDELLt et al 0.1459 [0.1391, 0.1527] |
| Omitting Lugero et al\r\n 0.1458 [0.1390, 0.1526] |
| Omitting Antonio Grimaldi et al 0.1456 [0.1388, 0.1525] |
| Omitting John Ellis et al 0.1465 [0.1397, 0.1533] |
| Omitting Scheel A et al 0.1490 [0.1421, 0.1558] |
| Omitting Beaton et al 0.1495 [0.1426, 0.1563] |
| Omitting J Freers et al 0.1471 [0.1403, 0.1539] |
| Omitting Ploutz M et al 0.1484 [0.1415, 0.1552] |
| Omitting Hovis et al 0.1491 [0.1423, 0.1560] |
| Omitting Gleason et al. 0.1476 [0.1407, 0.1544] |
| Omitting Rwebembera et al 0.1444 [0.1377, 0.1512] |
| **Pooled estimate 0.1467 [0.1399, 0.1535]** |
