# Supplementary material for: Rheumatic Heart Disease in East Africa: A Systematic Review and Meta-Analysis
Source: Int J Rheumatol. 2023 Sep 19;2023:8834443. doi: 10.1155/2023/8834443 (PMC10522432; doi:10.1155/2023/8834443)
Supplement: Supplementary Materials — The supplementary materials for the systematic review and meta-analysis of this review include Appendix 1 for the search strategy and information sources, Appendix 2 for the forest plot of the overall pooled prevalence of RHD in East Africa, Appendix 3 for the sensitivity analysis of the included studies, File 1 for the PRISMA-P of the preferred reporting items for systematic reviews and meta-analysis protocol, and File 2 for the Newcastle-Ottawa scale for the methodological quality assessment of the included studies. [file 8834443.f1.zip › Appendix 4.pdf]

Appendix 4: Forest plot for the sensitivity analysis of the included studies in the systematic review and meta analysis of rheumatic heart disease prevalence in East Africa

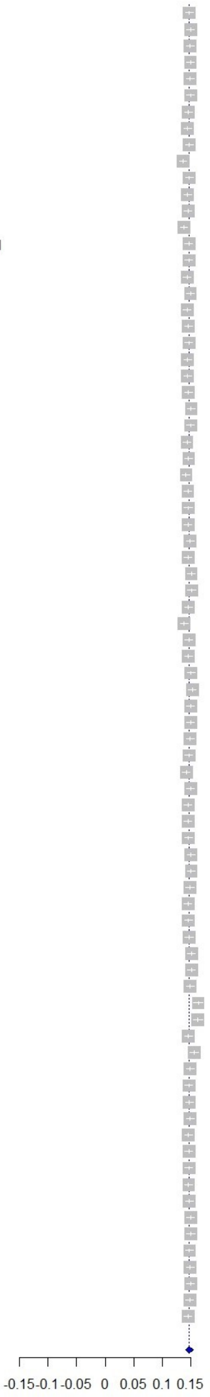

Study	Proportion	95%-CI	P-value	Tau2	Tau	I2
Omitting Massoure P.L et al	0.1466	[0.1398; 0.1534]	.	0.0007	0.0256	100%
Omitting Pierre-Laurent Massoure et al	0.1489	[0.1420; 0.1557]	.	0.0007	0.0257	100%
Omitting Rossi E et al	0.1484	[0.1416; 0.1553]	.	0.0007	0.0257	100%
Omitting Havard Otto et al	0.1488	[0.1419; 0.1556]	.	0.0007	0.0257	100%
Omitting W. TEWODROS et al	0.1482	[0.1413; 0.1550]	.	0.0007	0.0257	100%
Omitting Dawit Bacha et al	0.1488	[0.1419; 0.1556]	.	0.0007	0.0257	100%
Omitting Amir adem et al	0.1453	[0.1385; 0.1521]	.	0.0007	0.0256	100%
Omitting Richar M.Hodes	0.1436	[0.1368; 0.1504]	.	0.0007	0.0255	100%
Omitting N. Navuluri et al	0.1467	[0.1399; 0.1535]	.	0.0007	0.0257	100%
Omitting Senbeta Guteta et al	0.1358	[0.1293; 0.1424]	.	0.0006	0.0246	100%
Omitting Tefera et al.	0.1468	[0.1400; 0.1537]	.	0.0007	0.0256	100%
Omitting E. H. 0. PARRY	0.1439	[0.1371; 0.1507]	.	0.0007	0.0255	100%
Omitting T Hailemariam	0.1448	[0.1380; 0.1516]	.	0.0007	0.0256	100%
Omitting Yadeta D et al	0.1374	[0.1311; 0.1438]	.	0.0006	0.0236	100%
Omitting Gobezie Temesgen Tegegne et. al	0.1460	[0.1392; 0.1528]	.	0.0007	0.0257	100%
Omitting Tamirat Moges et al	0.1460	[0.1392; 0.1528]	.	0.0007	0.0257	100%
Omitting Hodes RM et al	0.1433	[0.1365; 0.1501]	.	0.0007	0.0255	100%
Omitting Mark E Engel et al	0.1488	[0.1419; 0.1556]	.	0.0007	0.0257	100%
Omitting Maru M. et al	0.1433	[0.1365; 0.1501]	.	0.0007	0.0255	100%
Omitting B Nigussie and H Tadele	0.1451	[0.1383; 0.1519]	.	0.0007	0.0256	100%
Omitting Gobezie Temesgen Tegegn et al.	0.1461	[0.1393; 0.1529]	.	0.0007	0.0257	100%
Omitting Tedros M. et al	0.1435	[0.1367; 0.1503]	.	0.0007	0.0255	100%
Omitting Belete H. et al	0.1438	[0.1370; 0.1505]	.	0.0006	0.0255	100%
Omitting Amare et al.	0.1452	[0.1384; 0.1520]	.	0.0007	0.0256	100%
Omitting Mulatu et al	0.1500	[0.1431; 0.1570]	.	0.0007	0.0260	100%
Omitting D. Yadeta et al	0.1492	[0.1423; 0.1560]	.	0.0007	0.0258	100%
Omitting Abebe Bekele et al	0.1427	[0.1363; 0.1492]	.	0.0006	0.0238	100%
Omitting abraha hailu et al	0.1455	[0.1387; 0.1523]	.	0.0007	0.0257	100%
Omitting Daniel E. et al.	0.1410	[0.1343; 0.1478]	.	0.0006	0.0253	100%
Omitting yusuf Mehadi et al.	0.1442	[0.1374; 0.1510]	.	0.0007	0.0256	100%
Omitting Solmon Gebremariam	0.1449	[0.1381; 0.1517]	.	0.0007	0.0256	100%
Omitting Ephrem D et al.	0.1448	[0.1380; 0.1516]	.	0.0007	0.0256	100%
Omitting Gemechu et Al.	0.1481	[0.1413; 0.1550]	.	0.0007	0.0257	100%
Omitting Tirfe et al.	0.1445	[0.1377; 0.1513]	.	0.0007	0.0256	100%
Omitting Oli k et al.	0.1502	[0.1432; 0.1571]	.	0.0007	0.0261	100%
Omitting Oli k et al.	0.1511	[0.1441; 0.1581]	.	0.0007	0.0265	100%
Omitting tamirat Moges et al	0.1455	[0.1387; 0.1523]	.	0.0007	0.0257	100%
Omitting Rebecca H. Lumsden et al.	0.1376	[0.1309; 0.1442]	.	0.0006	0.0247	100%
Omitting G O Oyoo et al.	0.1459	[0.1391; 0.1527]	.	0.0007	0.0257	100%
Omitting MYRA M. KOECH et al.	0.1450	[0.1382; 0.1518]	.	0.0006	0.0253	100%
Omitting Anabwani GM et al.	0.1498	[0.1429; 0.1567]	.	0.0007	0.0259	100%
Omitting G.M. Anabwani et al.	0.1521	[0.1450; 0.1592]	.	0.0007	0.0268	100%
Omitting Cosimo Marco Campanale et al.	0.1489	[0.1421; 0.1558]	.	0.0007	0.0257	100%
Omitting Cosimo Marco Campanale et al.	0.1491	[0.1422; 0.1559]	.	0.0007	0.0257	100%
Omitting Cosimo Marco Campanale et al.	0.1485	[0.1417; 0.1554]	.	0.0007	0.0257	100%
Omitting Neil Kennedy et al.	0.1459	[0.1391; 0.1527]	.	0.0007	0.0257	100%
Omitting Soliman EZ et al	0.1413	[0.1347; 0.1480]	.	0.0006	0.0247	100%
Omitting Sanyahumbi et al.	0.1488	[0.1420; 0.1557]	.	0.0007	0.0257	100%
Omitting Eberly et al	0.1443	[0.1375; 0.1511]	.	0.0007	0.0255	100%
Omitting Eberly et al	0.1448	[0.1380; 0.1516]	.	0.0007	0.0256	100%
Omitting Eberly et al	0.1450	[0.1382; 0.1518]	.	0.0007	0.0256	100%
Omitting J Mucumbitsi et al	0.1497	[0.1428; 0.1566]	.	0.0007	0.0259	100%
Omitting Sulafa Ali	0.1501	[0.1432; 0.1570]	.	0.0007	0.0261	100%
Omitting Ahmed AA Suliman	0.1479	[0.1410; 0.1547]	.	0.0007	0.0257	100%
Omitting Halm et al	0.1447	[0.1379; 0.1515]	.	0.0007	0.0255	100%
Omitting A I el Hag et al	0.1450	[0.1382; 0.1518]	.	0.0007	0.0256	100%
Omitting Musa et al	0.1464	[0.1396; 0.1532]	.	0.0007	0.0257	100%
Omitting Suha Khalil et al	0.1503	[0.1434; 0.1573]	.	0.0007	0.0262	100%
Omitting Ali et al	0.1505	[0.1435; 0.1574]	.	0.0007	0.0262	100%
Omitting Sulafa KM Ali et al	0.1480	[0.1411; 0.1548]	.	0.0007	0.0256	100%
Omitting J. Gupta et al	0.1623	[0.1542; 0.1704]	.	0.0010	0.0316	100%
Omitting Aly A et al	0.1620	[0.1539; 0.1701]	.	0.0010	0.0314	100%
Omitting kamal yassin et al	0.1451	[0.1383; 0.1519]	.	0.0007	0.0256	100%
Omitting Ibrahim-Khalil et al	0.1548	[0.1475; 0.1622]	.	0.0008	0.0280	100%
Omitting Zuechner et al	0.1481	[0.1412; 0.1549]	.	0.0007	0.0256	100%
Omitting PILLY CHILLO et al	0.1461	[0.1393; 0.1529]	.	0.0007	0.0257	100%
Omitting Makubi et al	0.1469	[0.1401; 0.1538]	.	0.0007	0.0257	100%
Omitting Paschal.J.Luggajo et al	0.1482	[0.1414; 0.1550]	.	0.0007	0.0257	100%
Omitting P. G. D'ARBELA et al	0.1452	[0.1384; 0.1520]	.	0.0007	0.0256	100%
Omitting JOAN L. CADDELLt et al	0.1459	[0.1391; 0.1527]	.	0.0007	0.0257	100%
Omitting Lugero et al	0.1458	[0.1390; 0.1526]	.	0.0007	0.0257	100%
Omitting Antonio Grimaldi et al	0.1456	[0.1388; 0.1525]	.	0.0007	0.0257	100%
Omitting John Ellis et al	0.1465	[0.1397; 0.1533]	.	0.0007	0.0257	100%
Omitting Scheel A et al	0.1490	[0.1421; 0.1558]	.	0.0007	0.0257	100%
Omitting Beaton et al	0.1495	[0.1426; 0.1563]	.	0.0007	0.0259	100%
Omitting J Freers et al	0.1471	[0.1403; 0.1539]	.	0.0007	0.0257	100%
Omitting Ploutz M et al	0.1484	[0.1415; 0.1552]	.	0.0007	0.0257	100%
Omitting Hovis et al	0.1491	[0.1423; 0.1560]	.	0.0007	0.0257	100%
Omitting Gleason et al.	0.1476	[0.1407; 0.1544]	.	0.0007	0.0257	100%
Omitting Rwebembera et al	0.1444	[0.1377; 0.1512]	.	0.0006	0.0252	100%
**Random effects model**	**0.1467**	**[0.1399; 0.1535]**	**.**	**0.0007**	**0.0257**	**100%**
