# Supplementary figures and images for: Rheumatic Heart Disease in East Africa: A Systematic Review and Meta-Analysis
Source: Int J Rheumatol. 2023 Sep 19;2023:8834443. doi: 10.1155/2023/8834443 (PMC10522432; doi:10.1155/2023/8834443)

## Appendix 2: Forest plot of the pooled prevalence of rheumatic heart disease in East Africa

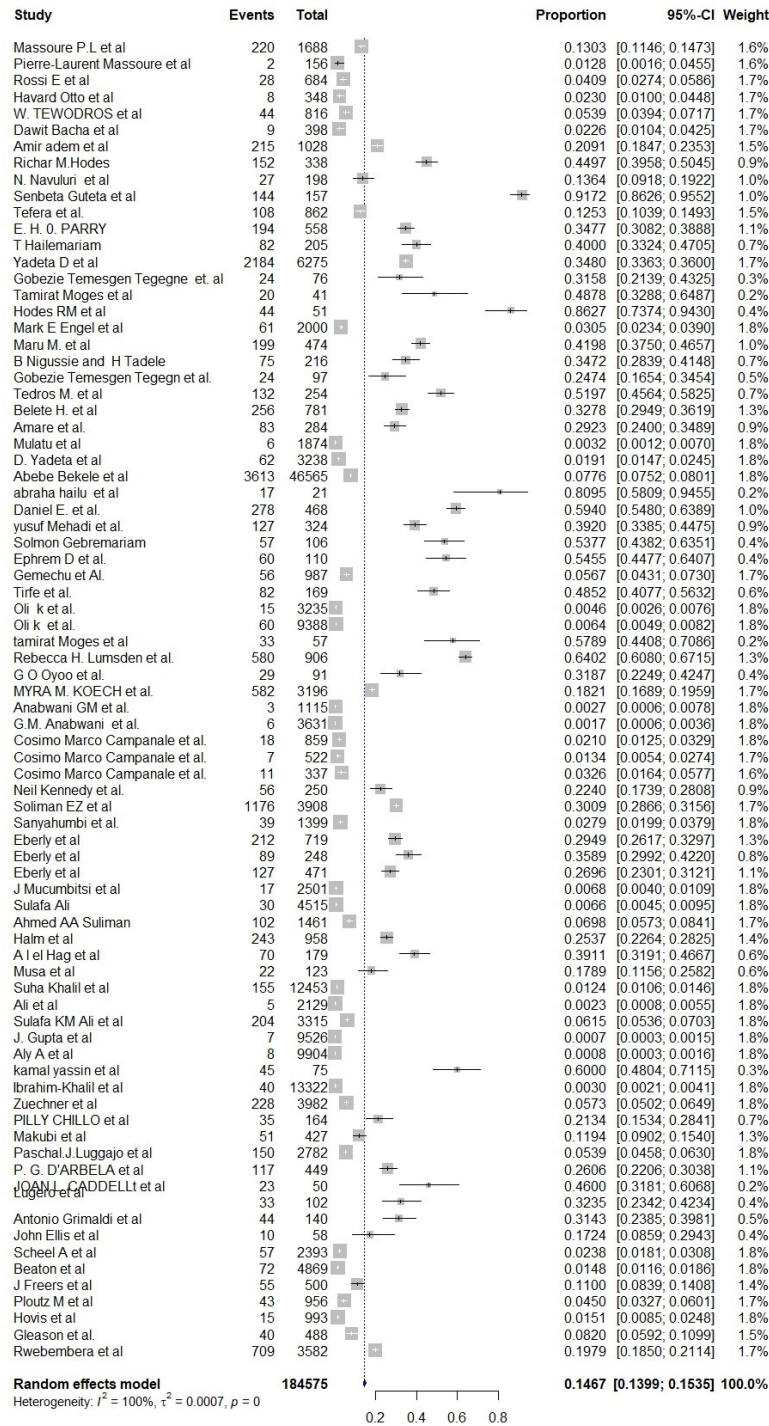

Supplement: Supplementary Materials — The supplementary materials for the systematic review and meta-analysis of this review include Appendix 1 for the search strategy and information sources, Appendix 2 for the forest plot of the overall pooled prevalence of RHD in East Africa, Appendix 3 for the sensitivity analysis of the included studies, File 1 for the PRISMA-P of the preferred reporting items for systematic reviews and meta-analysis protocol, and File 2 for the Newcastle-Ottawa scale for the methodological quality assessment of the included studies. [file 8834443.f1.zip › Appendix 2.pdf]
