# Supplementary material for: Rheumatic Heart Disease in East Africa: A Systematic Review and Meta-Analysis
Source: Int J Rheumatol. 2023 Sep 19;2023:8834443. doi: 10.1155/2023/8834443 (PMC10522432; doi:10.1155/2023/8834443)
Supplement: Supplementary Materials — The supplementary materials for the systematic review and meta-analysis of this review include Appendix 1 for the search strategy and information sources, Appendix 2 for the forest plot of the overall pooled prevalence of RHD in East Africa, Appendix 3 for the sensitivity analysis of the included studies, File 1 for the PRISMA-P of the preferred reporting items for systematic reviews and meta-analysis protocol, and File 2 for the Newcastle-Ottawa scale for the methodological quality assessment of the included studies. [file 8834443.f1.zip › File 1.doc]

**File 1. 2009 PRISMA checklist**

|  |  |  | **Reported** |
| --- | --- | --- | --- |
| **Section/topic** | **#** | **Checklist item** | **on page** |
|  |  |  | **#** |
|  |  |  |  |
| **TITLE** |  |  |  |
| Title | 1 | Identify the report as a systematic review, meta-analysis, or both. | 1 |
|  |  |  |  |
|  |  |  |  |
| **ABSTRACT** |  |  |  |
| Structured | 2 | Provide a structured summary including, as applicable: background; | 2 |
| summary |  | objectives; data sources; study eligibility criteria, participants, and |  |
|  |  | interventions; study appraisal and synthesis methods; results; limitations; |  |
|  |  | conclusions and implications of key findings; systematic review |  |
|  |  | registration number. |  |
|  |  |  |  |
|  | |  |  |
| **INTRODUCTION** | |  |  |
| Rationale | 3 | Describe the rationale for the review in the context of what is already | 3-4 |
|  |  | known. |  |
|  |  |  |  |
| Objectives | 4 | Provide an explicit statement of questions being addressed with reference | 4 |
|  |  | to participants, interventions, comparisons, outcomes, and study design |  |
|  |  | (PICOS). |  |
|  |  |  |  |
|  |  |  |  |
| **METHODS** |  |  |  |
| Protocol and | 5 | Indicate if a review protocol exists, if and where it can be accessed (e.g., | 8 |
| registration |  | Web address), and, if available, provide registration information including |  |
|  |  | registration number. |  |
|  |  |  |  |
| Eligibility | 6 | Specify study characteristics (e.g., PICOS, length of follow-up) and report | 5-6 |
| criteria |  | characteristics (e.g., years considered, language, publication status) used |  |
|  |  | as criteria for eligibility, giving rationale. |  |
|  |  |  |  |
| Information | 7 | Describe all information sources (e.g., databases with dates of coverage, | 4-5 |
| sources |  | contact with study authors to identify additional studies) in the search and |  |
|  |  | date last searched. |  |
|  |  |  |  |
| Search | 8 | Present full electronic search strategy for at least one database, including | 4-5 |
|  |  | any limits used, such that it could be repeated. |  |
|  |  |  |  |
| Study selection | 9 | State the process for selecting studies (i.e., screening, eligibility, included | 7 |
|  |  | in systematic review, and, if applicable, included in the meta-analysis). |  |
|  |  |  |  |
| Data collection | 10 | Describe method of data extraction from reports (e.g., piloted forms, | 7-8 |
| process |  | independently, in duplicate) and any processes for obtaining and |  |
|  |  | confirming data from investigators. |  |
|  |  |  |  |
| Data items | 11 | List and define all variables for which data were sought (e.g., PICOS, | 8 |
|  |  | funding sources) and any assumptions and simplifications made. |  |
|  |  |  |  |
| Risk of bias in | 12 | Describe methods used for assessing risk of bias of individual studies | 8 |
| individual |  | (including specification of whether this was done at the study or outcome |  |
| studies |  | level), and how this information is to be used in any data synthesis. |  |
|  |  |  |  |
| Summary | 13 | State the principal summary measures (e.g., risk ratio, difference in |  |
| measures |  | means). |  |
|  |  |  |  |
| Synthesis of | 14 | Describe the methods of handling data and combining results of studies, if | 8-9 |
| results |  | done, including measures of consistency (e.g., I^2^) for each meta-analysis. |  |
